# Effect of adjuvant chemotherapy on the oncological outcome of rectal cancer patients with pathological complete response

**DOI:** 10.1186/s12957-024-03300-0

**Published:** 2024-01-25

**Authors:** Jianguo Yang, Qican Deng, Yong Cheng, Zhongxue Fu, Xin Wu

**Affiliations:** 1 Department of General Surgery, The Third Affiliated Hospital of Chongqing Medical University, Chongqing, 401120 China; 2 Department of Gastrointestinal Surgery, The First Affiliated Hospital of Chongqing Medical University, Chongqing, 400016 China

**Keywords:** Rectal cancer, Pathological complete response, Adjuvant chemotherapy, Overall survival

## Abstract

**Background:**

Locally advanced rectal cancer is typically treated using a combination of neoadjuvant chemoradiotherapy and total mesorectal resection. While achieving pathological complete response following neoadjuvant chemoradiotherapy has been recognized as a positive prognostic factor in oncology, the necessity of adjuvant chemotherapy for locally advanced rectal cancer patients with pathological complete response after surgery remains uncertain. The objective of this meta-analysis was to examine the impact of adjuvant chemotherapy on the oncological outcomes of rectal cancer patients who attain pathological complete response after neoadjuvant chemoradiotherapy.

**Methods:**

This meta-analysis followed the guidelines outlined in the preferred reporting items for systematic review and meta-analysis (PRISMA). The Web of Science, PubMed, and Cochrane Library databases were systematically searched to identify relevant literature.

**Results:**

A total of 34 retrospective studies, including 9 studies from the NCBD database, involving 31,558 patients with pathological complete response rectal cancer, were included in the meta-analysis. The included studies were published between 2008 and 2023. The pooled analysis demonstrated that adjuvant chemotherapy significantly improved overall survival (HR = 0.803, 95% CI 0.678–0.952, *P* = 0.011), and no heterogeneity was observed (*I*^2^ = 0%). Locally advanced rectal cancer patients with pathological complete response who underwent adjuvant chemotherapy exhibited a higher 5-year overall survival rate compared to those who did not receive adjuvant chemotherapy (OR = 1.605, 95% CI 1.183–2.177, *P* = 0.002). However, the analysis also revealed that postoperative ACT did not lead to improvements in disease-free survival and recurrence-free survival within the same patient population. Subgroup analysis indicated that pathological complete response patients with clinical stage T3/T4, lymph node positivity, and younger than 70 years of age may benefit from adjuvant chemotherapy in terms of overall survival.

**Conclusions:**

The findings of this meta-analysis suggest that adjuvant chemotherapy has a beneficial effect on improving overall survival among rectal cancer patients with pathological complete response. However, no such association was observed in terms of disease-free survival and recurrence-free survival.

**Supplementary Information:**

The online version contains supplementary material available at 10.1186/s12957-024-03300-0.

## Introduction

The latest statistics on cancer in 2022 reveal that colorectal cancer (CRC) has emerged as a prominent cancer, ranking third in terms of incidence and second in mortality rates. It is worth noting that the prevalence of CRC is rapidly increasing [1]. Among all CRC cases, approximately 30% are attributed to rectal cancer, with a majority of cases being classified as locally advanced at the time of diagnosis [2]. The standard treatment approach for locally advanced rectal cancer (LARC) involves the utilization of neoadjuvant chemoradiotherapy (NCRT) combined with total mesorectal resection (TME). This treatment strategy offers multiple benefits, such as improved local tumor control, complete tumor removal, and sphincter preservation [3]. However, the response to NCRT in LARC patients varies considerably.

While a considerable proportion of LARC patients respond positively to NCRT, demonstrating tumor regression, only a relatively small percentage (ranging from 10 to 30% of cases) can achieve a pathological complete response (pCR) [4]. The achievement of pCR stands as a crucial milestone, indicating successful tumor eradication and favorable tumor biology. Extensive research has shown that patients who achieve pCR have remarkably low recurrence rates (6–17%) and high 5-year overall survival (OS) rates (87–92.9%) [5, 6]. A meta-analysis study revealed that patients with rectal cancer who attain pCR exhibit longer disease-free survival (DFS) and OS than those who do not achieve pCR [7]. Therefore, pCR is increasingly being recognized as a relevant endpoint in the design of clinical trials, acting as a surrogate marker for long-term tumor prognosis.

Adjuvant chemotherapy (ACT) is a commonly employed treatment modality for rectal cancer patients. However, there remains a lack of robust evidence regarding the use of ACT after NCRT and surgery. According to current National Comprehensive Cancer Network (NCCN) guidelines, all NCRT recipients should also undergo 6 months of ACT after surgery, regardless of their pathological regression response [8]. Nevertheless, the impact of ACT on OS and DFS among LARC patients who undergo NCRT is a subject of controversy. Some studies suggest that ACT may promote OS and DFS in LARC, while others contend that it does not affect the oncological prognosis of LARC patients who receive NCRT [9–11]. It is noteworthy that in several randomized controlled trials (RCTs) involving rectal cancer patients, the choice of postoperative systemic therapy is “at the discretion of the physician,” which contradicts the recommendations provided by the NCCN [12–14]. Despite the acknowledged prognostic advantage of achieving pCR in oncology, the necessity of ACT for LARC patients who attain pCR after surgery remains uncertain. Based on studies, some scholars argue that ACT improves OS in patients with pCR, while others assert that it may not be necessary for rectal cancer patients with pCR [15–19].

Therefore, the objective of this comprehensive meta-analysis was to investigate the impact of ACT on the oncological efficacy of LARC patients who achieved pCR after NCRT.

## Material and methods

In this study, we meticulously followed the guidelines set forth by the Preferred Reporting Items for Systematic Reviews and Meta-Analyses (PRISMA) [20]. By adhering to these rigorous standards, we aimed to ensure the credibility and integrity of the investigation’s findings (Table S2). This meta-analysis has been registered on the INPLASY platform with the registration number INPLASY2023120101 (https://inplasy.com/inplasy-2023-12-0101/).

### Literature search strategy

Two researchers performed an electronic literature search utilizing esteemed databases including Web of Science, PubMed, and Cochrane Library. The search was conducted until May 30, 2023. The search terms or keywords were as follows: [“Rectal cancer” OR “Rectal tumor” OR “Rectal neoplasm”] AND [“neoadjuvant radiotherapy” OR “neoadjuvant chemoradiation” “neoadjuvant chemoradiotherapy” OR “neoadjuvant treatment” OR “neoadjuvant therapy” OR “preoperative radiotherapy” OR “preoperative chemoradiation” OR “preoperative chemoradiotherapy” OR “preoperative treatment” OR “preoperative therapy”] AND [“adjuvant chemotherapy” OR “adjuvant therapy” OR “adjuvant treatment” OR “postoperative chemotherapy” OR “postoperative therapy” OR “postoperative treatment”] AND [“pathological complete response” OR “complete pathological response” OR “pCR” OR “pathological complete regression”]. Additionally, reference tracing was performed to minimize inadvertent exclusion of valuable studies. The detailed literature search strategy is shown in Table S3.

### Eligibility criteria

The search strategy was used to identify relevant studies from databases. Adhering to the PRISMA requirements, two researchers independently sifted through the trove of included studies. After removing duplicates, the researchers screened out studies based on titles and abstracts. Only those studies that satisfied the predetermined inclusion and exclusion criteria progressed to the next stage, where a comprehensive review of the full text ensued. The inclusion criteria were as follows: (1) patients with primary rectal cancer who received neoadjuvant chemoradiotherapy or radiotherapy; (2) adjuvant chemotherapy or observation after pCR; (3) radical surgery (APR, AR, Hartmann, ISR); and (4) outcomes including multivariate estimates value (HR, 95% CI) of OS, DFS or recurrence-free survival (RFS) or 5-year OS, DFS, or RFS rates. The exclusion criteria were as follows: (1) local excision or watch-and-wait patients; (2) no desired outcome reported; (3) neoadjuvant chemotherapy only; (4) ypT0 patients with unknown lymph node status; and (5) abstracts, meta-analyses, reviews, comments, and letters. LARC was defined as cT3/4, N0, M0 or cTx, N1-2, and M0 rectal cancer at initial diagnosis. pCR was defined as the absence of tumor cells in the primary tumor and lymph nodes after neoadjuvant therapy (ypT_0_N_0_M_0_). DFS was defined as the time from the date of surgery to the detection of disease relapse or death. RFS was defined as the time from the date of surgery to disease relapse (local or distant metastases). OS was defined as the time from the date of surgery to the date of death from any cause.

### Data extraction and quality assessment

The information was extracted from the full text according to a standardized form. The extracted information included general information such as authors, date of publication, source of data, and time period of the study. Basic clinical characteristics such as age, sex, clinical stage, neoadjuvant radiotherapy regimen, concurrent chemotherapy regimen, interval between last radiation and surgery, surgical modality, adjuvant chemotherapy, and duration of follow-up were also recorded. Oncological outcomes such as OS, DFS, and RFS were also recorded. To ensure the reliability and credibility of the retrospective cohort studies, the quality and methodology were assessed using the Newcastle–Ottawa Scale (NOS) score, which encompasses patient selection (4 points), cohort comparability (2 points), and evaluation of exposure or outcome (3 points) [21]. A score of 4 to 6 indicates moderate quality, while a score of 7 to 9 indicates high quality. All processes, including data extraction and NOS scoring, were carried out independently by two authors and meticulously cross-checked. In instances of disagreements, a third individual was consulted, allowing for robust discussions and the eventual attainment of a consensus.

### Statistical analysis

The primary focus was on hazard ratios (HRs) for OS, whereas secondary outcomes involved HRs for DFS and RFS. In addition, the researchers meticulously examined the 5-year rates of OS, DFS, and RFS. The HR and 95% confidence interval (CI) were considered the most appropriate statistic for evaluating the time-to-event outcomes of OS, DFS, and RFS. In cases where direct HR values for OS, DFS, and RFS were not available, they were estimated using Kaplan–Meier (KM) curves. Precision in estimating HR values was ensured through the employment of the eminent Parmar et al. and Tierney et al. specificity algorithms [22, 23]. Odds ratios (ORs) emerged as the outcome effect indicators, shedding light on the 5-year rates of OS, DFS, and RFS. Furthermore, the researchers utilized subgroup analyzes to explore age, clinical T-stage, and lymph node status as potential drivers of heterogeneity. The data were pooled and analyzed using STATA software (ver. 15; Stata Corp., College Station, TX, USA), and the results were presented using forest plots. Statistical heterogeneity was assessed using the *I*^2^ and Cochrane Q tests. If the* p* value exceeded 0.1 and *I*^2^ was below the 50%, it indicated that the heterogeneity was not significant, and a fixed-effect model was employed in this analysis. Conversely, statistical heterogeneity was recognized when the *p* value was below 0.1 or *I*^2^ exceeded 50%; the random-effects model was selected [24]. Sensitivity analyses were conducted to evaluate the reliability of the findings, while subgroup analyses were carried out to identify potential sources of heterogeneity. Funnel plots and Egger’s test were utilized to assess publication bias in the analyses of OS, DFS, and RFS [25]. Additionally, adjusted effect sizes were calculated using subtractive complementation if significant publication bias was detected. A statistical significance level of *p* < 0.05 was adopted.

## Results

### Literature selection and characteristics

**Fig. 1:**
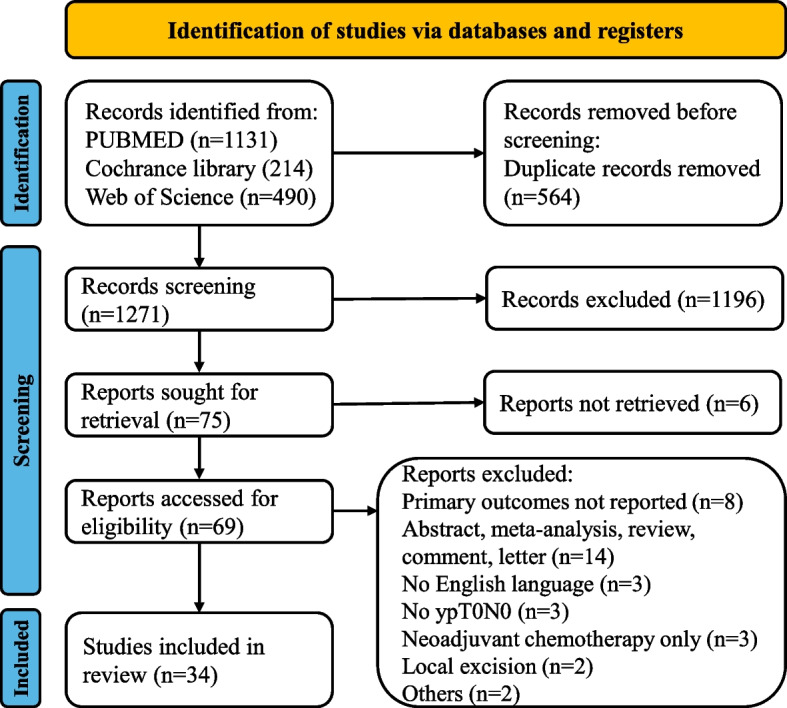
The flow diagram of PRISMA

Based on the subject terms, a total of 1835 articles were retrieved from various sources, including PubMed (*n* = 1131), Web of Science (*n* = 490), and Cochrane Library (*n* = 214). After removing 564 duplicate articles, we were left with 1271 potential articles. Upon reviewing the titles and abstracts, we were able to exclude 1196 articles that failed to meet the inclusion criteria. After careful examination of the full texts, 35 articles were further excluded for a multitude of reasons, such as failure to report primary outcomes (*n* = 8), being abstracts, meta-analyses, reviews, commentaries, or letters (*n* = 14), lacking English language (*n* = 3), lacking ypT_0_N_0_ (*n* = 3), having only neoadjuvant chemotherapy (*n* = 3),undergoing local excision (*n* = 2), or other reasons (*n* = 2). Eventually, a total of 34 [15–19, 26–54]. retrospective studies were included in the meta-analysis (Fig. 1).

The included studies were published between 2008 and 2023, with 9 [18, 26, 29, 31, 35, 38, 39, 43, 47]. of them sourced from the NCBD database. Among these studies, 15 were contributed by the USA, and 9 originated from China. In total, the meta-analysis included 31,558 rectal cancer patients who achieved pCR after nCRT. Out of these patients, 11,804 received postoperative ACT, while 19,754 underwent only observation and follow-up after radical surgery. The neoadjuvant therapy regimen commonly mentioned in studies consisted of long-course radiotherapy (45–54.5 Gy) along with concurrent chemotherapy using 5-Fu/capecitabine. The specific details about the included literature are presented in Table 1. The methodological quality of the retrospective studies was evaluated using the NOS scale, and all studies scored between 5 and 8 points. Among them, 9 studies scored 5 points (Table S1). Hence, the included studies exhibited an acceptable risk of bias.

**Table 1:** Basic characteristics of the included studies

Study	Year	Country	Type	Source of data	Duration	Sex (F:M)	Age	RT regime (Gy)	Concurrent chemotherapy	ACT regime	Clinical TNM stage	Interval between radiotherapy and operation (week)	No of patients		Follow-up time (month)	Outcomes	NOS score
													ACT	Non-ACT			
Chen et al. [17]	2023	China	Retrospective	Single center	2011–2019	1:1.6	Median: 53.1	46–50.4	FOLFOX	mFOLFOX6	II = 88, III = 192	NA	207	73	Mean: 55	OS, DFS	6
Lai et al. [19]	2023	USA	Retrospective	Multi-center	2004–2017	1:1.5	Median: 60.59	50–50.4	NA	NA	II = 1083, III = 1138	NA	780	1441	Median: 50.9	OS	7
Bliggenstorfer et al. [26]	2022	USA	Retrospective	NCDB	2010–2016	1:1.7	Mean: 60.21	45–54	NA	NA	II = 4524, III = 3418	NA	494	1054	NA	OS	6
Fukui et al. [27]	2022	Japan	Retrospective	Multi-center	2010–2017	NA	NA	45/50.4	5-FU	FL, FOLFOX	NA	6–8	75	30	Median:49	RFS	5
Kuo et al. [28]	2022	China	Retrospective	NHIRD and TCR	2007–2017	NA	NA	Median: 50.4	5-FU, capecitabine, oxaliplatin, leucovorin, UFUR	FL, capecitabine, FOLFOX, CapeOX, 5-FU + oxaliplatin	NA	NA	115	155	Median: 50.88	OS, DFS	5
Naffouje et al. [29]	2022	USA	Retrospective	NCDB	2004–2018	1:1.5	Mean: 57.2	45	NA	NA	II = 1123, III = 1461	5–12	1292	1292	Median: 56.4	OS	8
Jiang et al. [30]	2021	China	Retrospective	Single center	2009–2017	1:2	NA	48–55	Capecitabine, CapeOX	CapeOX, capecitabine	II = 67, III = 180	5–12	187	60	Median: 53	OS, RFS	6
Morris et al. [18]	2021	USA	Retrospective	NCDB	2006–2015	1:1.6	Median: 60.64	Median: 45	NA	NA	II = 1233, III = 1188	NA	778	1643	Median:42.3	OS	8
Gahagan et al. [31]	2020	USA	Retrospective	NCDB	2006–2013	1:1.6	Mean: 59.83	NA	NA	NA	II, III	NA	1513	4319	NA	OS	7
He et al. [16]	2020	China	Retrospective	Single center	2010–2018	1:1.9	Median: 55	50.4	Ora/ i.v. fluoropyrimidine	Capecitabine, FL, CapeOX, FOLFOX, FOLFOXIRI, FOLFIRI	II = 229, III = 780	4–8	712	297	Median: 35	OS, DFS, RFS	8
Voss et al. [32]	2020	USA	Retrospective	Multi-center	2005–2016	NA	NA	NA	5-FU, capecitabine, FOLFOX, CapeOX	Capecitabine, 5-FU, CapeOX, FOLFOX, oxaliplatin	NA	NA	139	54	Mean: 63	RFS	5
Hu et al. [33]	2019	China	Retrospective	Single center	2006–2016	1:1.7	Mean: 56.5	50	Capecitabine, CapeOX	CapeOX, Capecitabine	II = 55, III = 116	NA	56	115	11–138	OS, DFS	7
Nguyen et al. [34]	2019	USA	Retrospective	Single center	2000–2015	1:2	Mean: 58.14	NA	5-FU, capecitabine	Capecitabine, FOLFOX	II = 25, III = 71	Mean: 7	60	36	Mean: 77.76	OS, DFS	8
Dossa et al. [35]	2018	USA	Retrospective	NCDB	2006–2012	1:1.3	Median: 56.5	45–54	NA	NA	NA	< 9 = 927, ≥ 9 = 344	667	667	Median: 36.9	OS	8
Lu et al. [36]	2018	China	Retrospective	Multi-center	2005–2014	NA	NA	42–50	CapeOX, capecitabine	CapeOX, capecitabine, FOLFOX, oxaliplatin + S-1	NA	Median: 7.7	22	29	Median: 50	OS, RFS	6
Peng et al. [37]	2018	China	Retrospective	Single center	2008–2014	1:2	Mean: 52.9	46–50	CapeOX	CapeOX	II = 35, III = 70	6–8	83	22	Median: 49	OS, DFS	7
Polanco et al. [38]	2018	USA	Retrospective	NCDB	2006–2012	1:1.4	NA	NA	NA	NA	II = 698, III = 784	NA	741	741	Median: 39	OS	7
Turner et al. [39]	2018	USA	Retrospective	NCDB	2006–2011	1:1.6	Mean: 57.7	NA	NA	NA	II = 2183, III = 1922	NA	1379	2726	NA	OS	8
Gamaleldin et al. [40]	2017	USA	Retrospective	Single center	2000–2012	1:1.8	Mean: 58.9	Median: 50.4	5-FU, FL	NA	II = 73, III = 56	NA	47	83	Mean: 68.4	OS, DFS, RFS	5
Lichthardt et al. [41]	2017	Germany	Retrospective	Single center	1992–2013	NA	NA	NA	NA	5-FU, capecitabine, FOLFOX, FOLFIRI	NA	NA	9	15	NA	OS	5
Lorenzon et al. [42]	2017	Italy	Retrospective	Multi-center	2005–2015	NA	NA	50.4–56	Oral/i.v. fluoropirymidine	NA	NA	NA	77	155	Mean:47.6	OS	6
Shahab et al. [43]	2017	USA	Retrospective	NCDB	2006–2013	1:1.5	Mean: 60.1	NA	NA	NA	II = 1612, III = 1279	NA	789	2102	NA	OS	8
Kim et al. [44]	2017	Korea	Retrospective	Single center	2001–2013	NA	NA	45–50.4	FL, capecitabine	FL, capecitabine	NA	NA	50	40	Mean: 70.7	OS, DFS	6
Kuan et al. [45]	2016	China	Retrospective	TCR	2007–2013	1:1.7	Mean: 59.59	40–60	FL, tegafur, capecitabine	NA	II = 87, III = 172	≤ 8 = 173, > 8 = 86	114	145	Median: 37	OS	6
Tay et al. [46]	2016	Australia	Retrospective	ACCORD	2003–2014	NA	NA	50	Oral/i.v. fluoropyrimidine	Capecitabine, FOLFOX, FL	NA	NA	97	29	Median:45.5	OS, RFS	6
Xu et al. [47]	2016	USA	Retrospective	NCDB	2006–2011	NA	NA	NA	NA	NA	II, III	NA	484	1243	NA	OS	6
Zhou et al. [48]	2016	China	Retrospective	Single center	2005–2013	1:1.4	Mean: 54.05	50	CapeOX, FOLFOX4, capecitabine	CapeOX, FOLFOX4, capecitabine	II = 13, III = 22, Other = 5	NA	19	21	Median: 57	DFS	7
Lee et al. [49]	2015	Korea	Retrospective	Single center	1999–2009	NA	NA	50.4	Capecitabine	Uracil-tegafur, doxifluridine, Capecitabine	NA	6–8	32	12	Median:60.5	OS, DFS	5
Mass et al. [50]	2015	Netherlands	Retrospective	Multi-center	NA	1:1.9	Mean: 61	45–50.4	FL, FOLFOX	5-FU, capecitabine, FL, CapeOX, FOLFOX	NA	NA	290	608	NA	OS, DFS, RFS	6
Gave et al. [51]	2014	Israel	Retrospective	Single center	2001–2013	1:1.6	Median: 65.7	50.4	5-FU, capecitabine	NA	NA	Mean: 11.66	35	17	Mean: 49.4	OS, DFS	5
Kiran et al. [52]	2012	USA	Retrospective	Single center	2000–2008	NA	NA	50.4	5-FU, FL	NA	NA	NA	14	34	Median: 52.6	OS, DFS	6
Govindarajan et al. [53]	2011	USA	Retrospective	Single center	1999–2003	NA	NA	50.4	5-FU-based	FL, FOLFOX	II, III	4–8	64	9	Median:69.6	RFS	5
Yeo et al. [54]	2010	Korea	Retrospective	Single center	1993–2007	NA	NA	39.6–54	5-FU, FL, FOLFOX, FOLFIRI, capecitabine	Oral/i.v. fluoropyrimidine, FOLFIRI, FOLFOX	NA	Median: 6	256	48	Median: 43	DFS	6
Capirci et al. [15]	2008	Italy	Retrospective	Multi-center	1900–2004	1:1.9	Mean: 61.8	Mean: 50	5-FU, capecitabine, raltitrexed, 5-FU + Mitomycin C, 5-FU + cisplatin, oxaliplatin + 5-FU, oxaliplatin + raltitrexed, oxaliplatin + capecitabine	NA	I = 33, II = 250, III = 254, Unknow = 29	≤ 10 = 456, > 10 = 110	127	439	Median:45.6	OS, DFS	5

Note: *F* female, *M* male, *ACT* adjuvant chemotherapy, *NOS* Newcastle–Ottawa Scale, *NCBD* National Cancer Database, *TCRD* Taiwan Cancer Registry Database, *ACCORD* Australian Comprehensive Cancer Outcomes and Research Database, *NA* not available, *FOLFOX* folinic acid + fuorouracil + oxaliplatin, *FU* fuorouracil, *FL* fuorouracil + leucovorin, *CapeOX* capecitabine + oxaliplatin, *FOLFIRI* folinic acid + fuorouracil + irinotecan, *OS* overall survival, *DFS* disease-free survival, *RFS* recurrence-free survival

### The oncological outcome in pCR patients with or without ACT

#### Overall survival

**Fig. 2:**
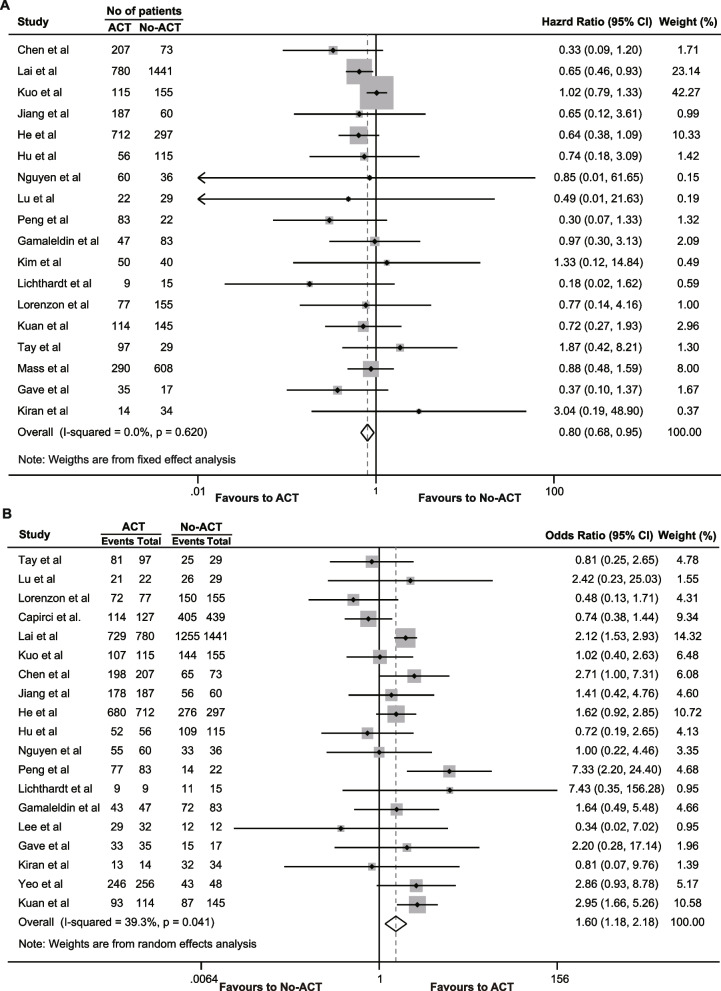
The effects of adjuvant chemotherapy on overall survival. **A** Hazard ratio of overall survival; **B** 5-year overall survival rate

A total of 29 [15–19, 26, 28–31, 33–52]. studies, including 9 [18, 26, 29, 31, 35, 38, 39, 43, 47]. from the NCBD database, provided reporting on OS. For the pooled analysis, we only included the most recently published studies from this database. Given the 18 [16, 17, 19, 28, 30, 33, 34, 36, 37, 40–42, 44–46, 50–52]. studies with reporting on the effect of ACT on the hazard ratio of OS in patients with rectal cancer in pCR, the pooled analysis showed that ACT improved overall survival (HR = 0.803, 95% CI 0.678–0.952, *P* = 0.011) without any observed heterogeneity (*I*^2^ = 0%, χ^2^ = 14.66, *P* = 0.620) (Fig. 2A). Additionally, 19 [15–17, 19, 28, 30, 33, 34, 36, 37, 40–42, 45, 46, 49, 51, 52, 54]. studies reported on the 5-year OS rate, and the analysis revealed that patients with pCR who underwent ACT had a higher 5-year OS rate than those who did not receive ACT (OR = 1.605, 95% CI 1.183–2.177, *P* = 0.002). There was moderate heterogeneity in the pooled analysis (*I*^2^ = 39.3%, χ^2^ = 29.68, *P* = 0.041), so a random-effects model was used (Fig. 2B). To address potential bias from duplicated patient data in the NCDB cohort, we conducted separate pooled analyses for each study in the NCBD database. These separate analyses also indicated that postoperative ACT improved the OS in patients with pCR (Table 2).

**Table 2:** The pooled analysis of OS from different NCBD database studies

Hazard ratio of OS						5-years OS rate					
Study	HR	95% CI		*I*^2^ (%)	*P*	Study	OR	95% CI		*I*^2^	*P*
		LCI	UCI					LCI	UCI		
All [18, 26, 29, 31, 35, 38, 39, 43, 47]	0.65	0.57	0.74	46	< 0.001^*^	All [18, 26, 29, 31, 35, 38, 39, 43]	1.890	1.623	2.198	49.8	< 0.001^*^
Lai et al. [19]	0.803	0.678	0.952	0	0.011	Lai et al [19]	1.605	1.182	2.179	39.3	0.002^*^
Bliggenstorfer et al. [26]	0.756	0.633	0.903	25.8	0.002	Bliggenstorfer et al. [26]	1.626	1.179	2.242	43	0.003^*^
Naffouje et al. [29]	0.812	0.716	0.921	0	0.001	Naffouje et al. [29]	1.543	1.159	2.053	34.9	0.003^*^
Morris et al. [18]	0.776	0.658	0.914	0	0.002	Morris et al. [18]	1.600	1.185	2.160	38.7	0.002^*^
Gahagan et al. [31]	0.790	0.694	0.9	0	< 0.001	Gahagan et al. [31]	1.546	1.175	2.033	34.9	0.002^*^
Dossa et al. [35]	0.774	0.648	0.925	13.9	0.005	Dossa et al. [35]	1.626	1.175	2.252	43	0.003^*^
Polanco et al. [38]	0.662	0.577	0.76	36	< 0.001	Polanco et al. [38]	1.616	1.176	2.222	41.1	0.003^*^
Turner et al. [39]	0.831	0.729	0.947	0	0.005	Turner et al. [39]	1.567	1.189	2.066	35.4	0.001^*^
Shahab et al. [43]	0.82	0.68	0.98	0	0.031	Shahab et al. [43]	1.650	1.168	2.331	52.1	0.004^*^
Xu et al. [47]	0.784	0.654	0.941	13.9	0.009						

*random effects model; *HR* hazard ratio, *OR* odds ratio, *CI* confidence interval, *LCI* low confidence interval, *UCI* upper confidence interval, *OS* overall survival

#### Disease-free survival

**Fig. 3:**
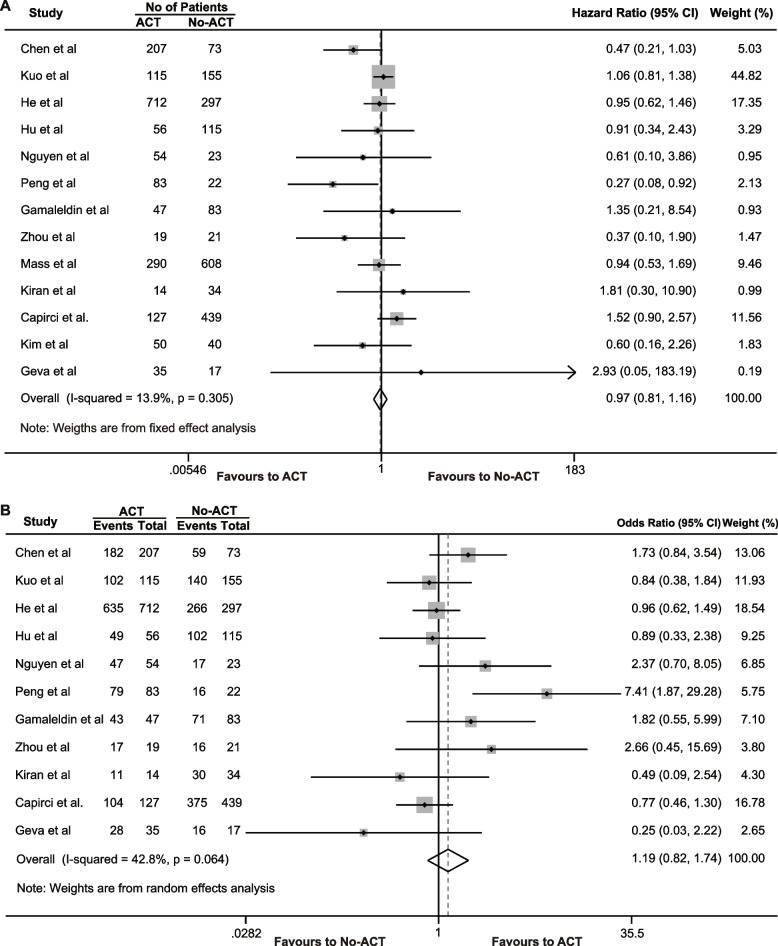
The effects of adjuvant chemotherapy on disease-free survival. **A** Hazard ratio of disease-free survival; **B** 5-year disease-free survival rate

Thirteen [15–17, 28, 33, 34, 37, 40, 44, 48, 50–52]. studies compared the effect of ACT and non-ACT on DFS in rectal cancer patients who achieved a pCR. The pooled analysis revealed that ACT did not have a significant impact on DFS in patients with pCR (HR = 0.97, 95% CI 0.81–1.16, *P* = 0.765), with only mild heterogeneity observed (*I*^2^ = 13.9%, χ^2^ = 13.94, *P* = 0.305) (Fig. 3A). Furthermore, 11 [15–17, 28, 33, 34, 37, 40, 48, 51, 52]. studies examined the effect of ACT on the 5-year DFS rates in patients with pCR. The results indicated that ACT also failed to improve the 5-year DFS rate in rectal cancer patients with pCR (OR = 1.192, 95% CI 0.818–1.736, *P* = 0.360), and there was moderate heterogeneity in the pooled analysis (*I*^2^ = 39.3%, χ^2^ = 29.68, *P* = 0.041) (Fig. 3B).

#### Recurrence-free survival

**Fig. 4:**
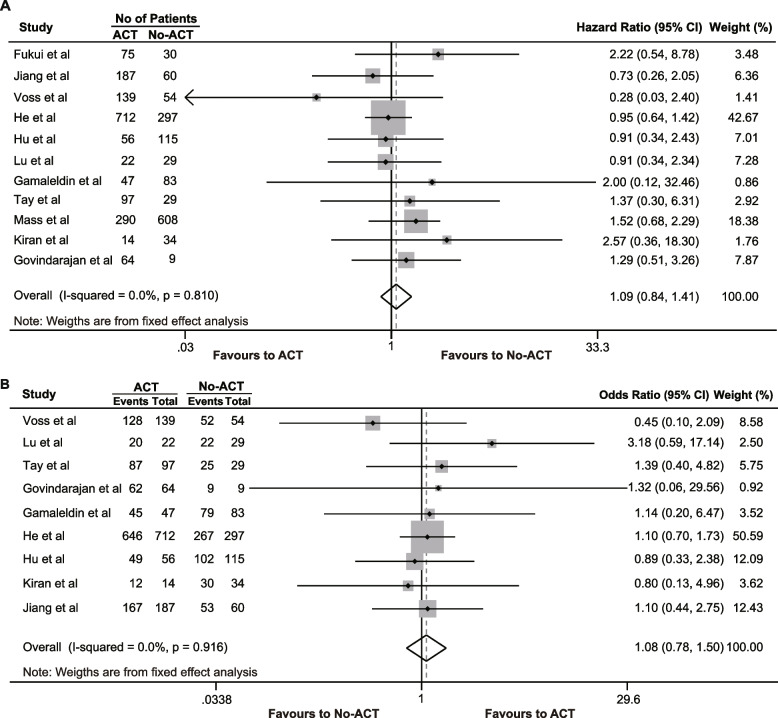
The effects of adjuvant chemotherapy on recurrence-free survival. **A** Hazard ratio of recurrence-free survival; **B** 5-year recurrence-free survival rate

We collected data on RFS from 11 [16, 27, 30, 32, 33, 36, 40, 46, 50, 52, 53]. studies, which indicated that the RFS of pCR patients who received ACT was similar to that of those who did not receive ACT (HR = 1.087, 95% CI 0.838–1.410, *P* = 0.531), and there was no heterogeneity among the studies (*I*^2^ = 0%, χ^2^ = 6.06, *P* = 0.810) (Fig. 4A). From the nine [16, 30, 32, 33, 36, 40, 46, 52, 53]. studies that included reporting of a 5-year RFS rate in pCR patients, the pooled results showed that ACT also did not improve the 5-year RFS rate (OR = 1.084, 95% CI 0.780–1.507, *P* = 0.630). No heterogeneity was observed (*I*^2^ = 0%, χ^2^ = 3.27, *P* = 0.916) (Fig. 4B).

#### Subgroup analysis

**Fig. 5:**
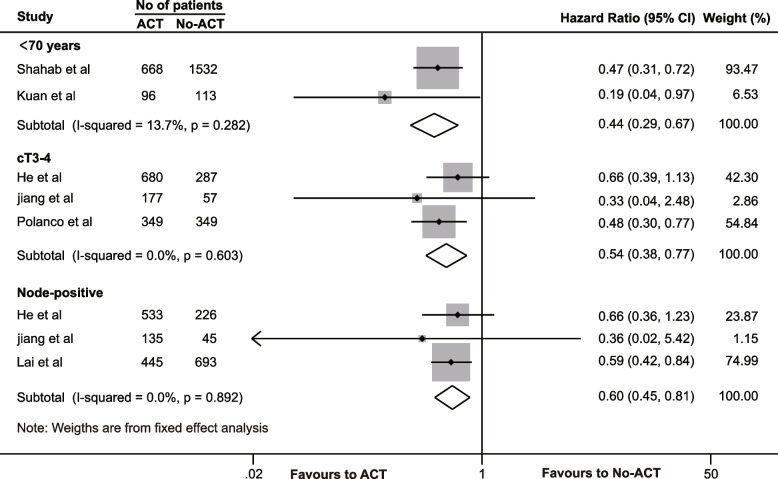
Subgroup analysis of oncological effects of adjuvant chemotherapy

Subgroup analysis revealed that ACT could enhance OS in patients younger than 70 years old [43, 45]. with rectal cancer who achieved a pCR (HR = 0.443, 95% CI = 0.295–0.666, *P* < 0.001). Furthermore, pCR patients with clinical stage T3/T4 [16, 30, 38]. or lymph node positivity [16, 19, 30]. also experienced improved OS with ACT (cT3/4, HR = 0.544, 95% CI = 0.384–0.771, *P* = 0.001; N^+^, HR = 0.603, 95% CI = 0.446–0.813, *P* = 0.001) (Fig. 5).

### Publication bias

Publication bias was assessed by visualizing the funnel plots. The data analysis indicated that the funnel plots were symmetrical for the included studies (Fig. S1). Furthermore, Egger’s test also supported the absence of publication bias in the pooled studies (OS, *P* = 0.242; DFS, *P* = 0.235; RFS, *P* = 0.628).

## Discussion

The objective of this meta-analysis was to investigate the influence of postoperative ACT on oncological outcomes in patients with rectal cancer who achieved pCR following NCRT. The pooled data indicated that ACT was ineffective in mitigating the hazard ratios relating to DFS and RFS in rectal cancer patients with pCR. However, rectal cancer patients with a pCR who received ACT exhibited enhanced OS within the same patient cohort.

The justification for routinely administering ACT for rectal cancer is primarily derived from extrapolating the survival benefits of ACT for colon cancer patients [55, 56]. Nevertheless, there is no direct evidence to corroborate that ACT after NCRT and TME surgery improves the prognosis of rectal cancer patients [9–11]. The NCCN guidelines advocate administering ACT for stage II/III rectal cancer with or without NCRT, irrespective of postoperative pathological stage [8]. Evidence supporting the use of ACT after NCRT and surgery is primarily based on the ability of postoperative chemotherapy or radiotherapy to ameliorate oncological outcomes in rectal cancer. For example, a meta-analysis of 20 randomized trials revealed that the utilization of fluorouracil-based ACT in the treatment of rectal cancer significantly promoted OS (HR 0.83, 95% CI 0.76–0.91) and DFS (HR 0.75, 95% CI 0.68–0.83) following surgical intervention [57]. Nevertheless, only one of the included randomized trials involved administering NCRT prior to the operation [13].According to the European Society for Medical Oncology (ESMO) guidelines, ACT is solely recommended for stage III rectal cancer or stage II rectal cancer with high-grade risk factors after NCRT [58]. The ESMO guidelines also indicated that the evidence for the effectiveness of ACT for rectal cancer patients with NCRT is much weaker in comparison to colon cancer. In rectal cancer, it is probable that ACT would have a limited effect on OS, but could enhance DFS after NCRT [55, 58]

In the EORTC 22921 trial, rectal cancer patients who underwent neoadjuvant chemoradiotherapy or radiotherapy were randomly assigned to receive either ACT (5-FU/LV) or an observation. The findings revealed that the Kaplan–Meier curves of DFS and OS seemed to diverge after 2 and 4 years, respectively, with a preference for the group receiving ACT. No benefit was observed in terms of OS (HR 0.91, 95% CI 0.77–1.09) or DFS (HR 0.91, 95% CI 0.77–1.08) in the ACT group after a median follow-up of 5.4 and 10.4 years, respectively [13, 59, 60]. The Dutch Proctor-SCRIPT trial and the I-CNR-RT trial also highlighted that ACT with 5-FU/LV or capecitabine did not improve DFS and OS in patients with rectal cancer when compared with observations alone [61, 62]. However, due to poor patient compliance with ACT, early termination of the trials caused by poor recruitment, and suboptimal chemotherapy regimens, the conclusions of these randomized trials remain questionable. A meta-analysis was conducted to investigate the impact of ACT on the OS and DFS of LARC patients after NCRT. The study showed that ACT considerably improved both OS and DFS in comparison to non-ACT. Additionally, the subgroup analysis revealed that ACT was especially beneficial for patients with node-negative or ypStage III LARC in terms of OS. In non-RCT, the pooled data indicated a marked rise in OS in the ACT group when contrasted with the observation group. However, upon isolating only randomized controlled studies, a significant variation in OS between the ACT and non-ACT groups was not observed [63].

The current clinical practice of administering ACT for rectal cancer following NCRT and surgery lacks consistency, particularly in patients who experience a pCR. Rectal cancer patients with pCR have demonstrated exceptional oncologic outcomes [5, 6]. This brings into question the necessity of ACT for rectal cancer patients with pCR and raises concerns regarding overtreatment. Several cohort studies from the NCBD database have retrospectively analyzed the prognosis of pCR rectal cancer patients with ACT or observation [18, 26, 29, 31, 35, 38, 39, 43, 47]. The results have consistently demonstrated that ACT is beneficial in improving OS in rectal cancer patients with pCR. However, two of the studies indicated that approximately 70% of rectal cancer patients who underwent NCRT and surgery did not receive ACT, which is divergent from the proportion of rectal cancer patients receiving ACT reported in the SEER database [35, 38, 64]. Furthermore, the significant advantage of ACT for rectal cancer patients with pCR is perplexing. There is a possibility that the effect of ACT for rectal cancer with pCR may be overestimated, although the patient subgroup was identified from the NCBD database during the same period to test the association of ACT with survival. Moreover, the number and proportion of rectal cancer patients with pCR varied considerably among studies. Although some studies showed benefits, other retrospective studies found no improvement in the OS of rectal cancer patients with pCR who received ACT [16, 17, 28, 42, 45, 50]. He et al. enrolled 1041 rectal cancer patients with pCR, of whom 303 patients did not receive ACT, while 738 patients received fluorouracil-based ACT. After propensity score matching, the analysis indicated that the adjuvant and non-ACT groups exhibited similar results in OS (HR = 1.558, 95% CI 0.92–2.64), DFS (HR = 1.05, 95% CI 0.68–1.62), local recurrence-free survival (HR = 1.01, 95% CI 0.30–2.60), and distant metastasis-free survival (HR = 1.06, 95% CI 0.68–1.64). Furthermore, there was no improvement observed in OS and DFS for pCR rectal cancer patients administrated different cycle ACT cycles (0 *vs.*1–4 *vs.* ≥ 5) [16]. A recent study performed a subgroup analysis to examine the oncological outcomes of pCR rectal cancer patients with acellular mucin pools. The findings revealed that pCR rectal cancer patients without acellular mucin pools had DFS compared to those with acellular mucin pools (*P* = 0.037). Furthermore, ACT was found to be associated with improved DFS (*P* = 0.003) and OS (*P* = 0.027) in pCR rectal cancer patients with acellular mucin pools. This could be attributed to the fact that the presence of acellular mucin pools may indicate tumor invasion, and for pCR patients with acellular mucin pools, ACT may be beneficial in eradicating any residual micrometastatic disease [17].Therefore, it is suggested that ACT and close follow-up are necessary for this particular subset of pCR rectal cancer patients with acellular mucin pools.

Prior meta-analyses examining the impact of ACT in rectal cancer patients with pCR have yielded conflicting results. Ma et al. conducted a meta-analysis and discovered that ACT significantly improved OS (HR = 0.65, 95% CI = 0.46–0.90) compared to observation alone in rectal cancer patients with pCR [65]. Lim et al. conducted a separate pooled analysis involving studies from different NCBD sources. They observed a tendency toward enhanced OS in pCR rectal cancer patients receiving ACT, irrespective of whether studies from a specific NCBD database were included in the statistical analysis of various subgroups [66]. However, this analysis did not consistently demonstrate any significant differences. Another meta-analysis of 23 non-randomized controlled studies also suggested that ACT promoted OS in pCR rectal cancer patients (HR = 0.68, 95% CI 0.55–0.84). Nevertheless, there was no marked advantageous effect on DFS or RFS. Six of these studies were taken from the NCBD database, and the existence of overlapping data has the potential to exaggerate the perceived benefit of ACT in rectal cancer patients with pCR, which could introduce bias in the overall analysis [67]. In comparison to the prior meta-analysis, we more comprehensively selected studies in this meta-analysis to permit a more reliable evaluation of the correlation between ACT and prognosis in pCR rectal cancer. We included ten studies sourced from the NCBD database, specifically opting for the most recently published studies to prevent duplication of data and ensure the precision of our findings. In addition, we meticulously summarized the data for each study obtained from the NCBD database individually to avoid duplication of information that could result in erroneous conclusions. We further performed subgroup analyses on factors that could affect tumor outcome, including age, lymph node status, and clinical T-stage. The aim was to ascertain how these variables influence the link between ACT and prognosis in pCR rectal cancer. These additions offer valuable insights into the relationship between ACT and prognosis in pCR rectal cancer patients.

Many factors can affect the oncological outcome of rectal cancer after NCRT and surgery, such as age, performance status, comorbidities, postoperative complications, colectomy, pathological TNM stage, and ACT [68–71]. ACT was more likely to be used in younger patients (age, < 60) and in individuals with better performance status [43]. It is well known that younger age and better performance status are favorable and independent prognostic factors for OS. In addition, patients with a younger age and a better performance status tend to be more compliant and tolerant toward ACT than their older counterparts with a poorer performance status. Hence, when rectal cancer patients belonging to the ACT cohort exhibit a younger age and better performance status, an overestimation of the effect of ACT on OS could result. Our meta-analysis indicated that ACT improved OS only among rectal cancer patients with pCR, but had no significant effect on DFS or RFS. A possible explanation is that the OS benefit as a whole could be attributed to younger age and better performance status, instead of ACT treatment. If ACT does have a benefit, it is likely to be minimal. The improvement in OS was driven predominantly by reductions in disease recurrence and cancer-related deaths. In addition, in the subgroup analysis of this study, ACT was capable of decreasing the hazard ratio of OS in pCR rectal cancer patients younger than 70 years. On the other hand, no benefit of ACT was observed in rectal cancer patients with pCR who were older than 70 years (Fig. S2). Owing to the absence of detailed data on individual patients, we were not able to explore the factors that influence OS and DFS in rectal cancer patients with pCR. Therefore, this result should be interpreted with caution.

In recent years, a novel treatment approach termed total neoadjuvant therapy (TNT) has been proposed to address the issue of poor compliance and tolerance to ACT in patients with rectal cancer [72, 73]. This involves strengthening the neoadjuvant therapy with induction or consolidation chemotherapy in conjunction with NCRT. Compared to conventional NCRT, TNT has shown improvements in the resectability rate and pCR rate for LARC. It also promotes compliance with systemic therapy and increases the percentage of patients who complete chemotherapy, thus boosting the probability of organ preservation [74–76]. The NCCN guidelines recommend TNT as a viable treatment alternative for rectal cancer patients diagnosed with T3 tumors exhibiting positive circumferential resection margins, T4 stage, positive lymph nodes, locally unresectable tumors, or those with a performance status that renders them unsuitable for surgery [8]. For pCR rectal cancer patients who are unable to receive ACT due to complications, colostomy, poor performance status, or chemotherapy intolerance, TNT can enhance their oncological outcomes. Nevertheless, the optimal radiotherapy regimen (long/short course radiotherapy), chemotherapy regimen, and the sequence between radiotherapy and chemotherapy (induction/consolidation chemotherapy) are still subjects of controversy that demand evaluation by an experienced multidisciplinary team before implementation [77, 78].

There were some limitations that need to be acknowledged in relation to this meta-analysis. First, the absence of prospective randomized controlled trials investigating the necessity of ACT for rectal cancer with pCR was a notable limitation. The studies encompassed in this analysis were solely retrospective cohort studies, characterized by varying sample sizes, baseline characteristics, and treatment protocols. Thus, the presence of information bias and confounding factors was inevitable. Additionally, certain studies only provided Kaplan–Meier curves, which needed the estimation of HRs and 95% CIs for OS, DFS, and RFS. It was crucial to acknowledge that such estimations extracted from Kaplan–Meier curves may stray from the original data, resulting in likely inaccuracies in the pooled data. Third, the considerable heterogeneity observed in the sample sizes of the included studies deserves attention. While 9 studies were derived from the NCDB, each of these studies exhibited a large sample size and yielded positive findings. This significant variability in sample sizes potentially led to an overestimation of the benefits associated with ACT. Furthermore, the limited number of studies reporting the impact of ACT on patients with pCR rectal cancer, stratified by age, clinical T stage, and lymph node status, is worth noting. The findings of our study suggested that patients younger than 70 years old, those with cT3/4 tumors, or those with lymph node-positive pCR rectal cancer may derive benefits from ACT. However, it is crucial to acknowledge that these conclusions were based on a small number of studies with inherent limitations. Moreover, it is important to recognize that all the included studies originated from the NCDB, potentially introducing selection and information biases. Therefore, considering the limitations identified within this study, it is imperative that further high-quality randomized controlled trials are conducted to validate the effects of ACT on the oncological prognosis of patients with pCR rectal cancer.

## Conclusion

In conclusion, the results of our meta-analysis suggested a beneficial effect of adjuvant chemotherapy in improving overall survival in rectal cancer patients with pathological complete response. However, this association was not observed in terms of disease-free survival and recurrence-free survival.

### Supplementary Information

**Additional file 1: Figure S1.** The funnel plot for publication bias. A. Overall survival; B. Disease-free survival; C. Recurrence-free survival, **Table S1.** The NOS score of included studies, **Table S2.** PRISMA 2020 Checklist, **Table S3.** The strategy of literature search.

## Data Availability

All data generated or analyzed during this study are included in this published article. Further inquiries can be directed to the corresponding author.

## Abbreviations

LARC: Locally advanced rectal cancer
NCRT: Neoadjuvant chemoradiotherapy
TME: Total mesorectal resection
pCR: Pathological complete response
ACT: Adjuvant chemotherapy
OS: Overall survival
DFS: Disease-free survival
RFS: Recurrence-free survival
CRC: Colorectal cancer
NOS: Newcastle-Ottawa Scale
HR: Hazard ratio
OR: Odds ratio
CI: Confidence interval
TNT: Total neoadjuvant therapy
RCT: Randomized controlled trials
